# Statement of Retraction: Role of dynorphin in memory deficits associated with chronic pain

**DOI:** 10.1080/24740527.2024.2337608

**Published:** 2024-04-15

**Article title**: “Role of dynorphin in memory deficits associated with chronic pain” in 2022 Research Poster Abstracts

**Authors**: Emma Mannan, Loren Martin

**Journal**: *Canadian Journal of Pain*

**Bibliometrics**: Volume 06, Issue 03, Page A127

**DOI**: http://dx.doi.org/10.1080/24740527.2022.2088027

Since publication, concerns have been raised about the integrity of the data reported as part of the abstract “Role of dynorphin in memory deficits associated with chronic pain” on page A127. Following an investigation by the University of Toronto, it was found the western blot data reported for this study could not be verified. Given the uncertainty about these data, the authors cannot confirm the accuracy of the rest of the abstract and therefore the corresponding author, the Canadian Pain Society, the Editor-in-Chief and Publisher are retracting the article from the journal.

